# Long-term clinical outcomes in cirrhotic chronic hepatitis B patients treated with tenofovir disoproxil fumarate for up to 5 years

**DOI:** 10.1007/s12072-015-9614-4

**Published:** 2015-03-13

**Authors:** Maria Buti, Scott Fung, Edward Gane, Nezam H. Afdhal, Robert Flisiak, Selim Gurel, John F. Flaherty, Eduardo B. Martins, Leland J. Yee, Phillip Dinh, Jeffrey D. Bornstein, G. Mani Subramanian, Harry L. A. Janssen, Jacob George, Patrick Marcellin

**Affiliations:** 1 Servei de Medicina Interna-Hepatologia, Hospital General Universitari Vall d’Hebron, Pg. Vall d’Hebron, 119-129, 08035 Barcelona, Spain; 2 Department of Medicine, Toronto General Hospital, University of Toronto, 200 Elizabeth Street, Toronto, ON M5G 2C4 Canada; 3 Department of Gastroenterology and Hepatology, Auckland City Hospital, 2 Park Road, Auckland, 1142 New Zealand; 4 Department of Hepatology, Beth Israel Deaconess Medical Center, 330 Brookline Avenue, Boston, MA 02215 USA; 5 Department of Infectious Diseases and Hepatology, Medical University of Białystok, 15-540 Białystok, Poland; 6 Department of Gastroenterology, University of Uludag, Özlüce Mh., 16120, Bursa, 16059 Turkey; 7 Gilead Sciences, Inc., 333 Lakeside Drive, Foster City, CA 94404 USA; 8 Division of Gastroenterology, Erasmus MC University Hospital, Rotterdam, The Netherlands; 9 Storr Liver Unit, Westmead Millennium Institute, Westmead Hospital, University of Sydney, Hawkesbury Road, Westmead, NSW 2145 Australia; 10 Service d’Hépatologie, Viral Hepatitis Research Centre, Hôpital Beaujon, 100 Boulevard du General Leclerc, 92110 Clichy, France

**Keywords:** Antiviral agent, Chronic hepatitis B, Cirrhosis, Hepatitis B e antigen, Hepatitis B surface antigen, Tenofovir disoproxil

## Abstract

**Background:**

Phase 3 clinical studies have shown that long-term treatment with tenofovir disoproxil fumarate (TDF) can suppress hepatitis B viral load and promote significant fibrosis regression and cirrhosis reversal in a majority of treated chronic hepatitis B (CHB) patients. This retrospective analysis investigated the impact of baseline cirrhosis status on virologic, serologic, and histologic outcomes in patients treated with TDF.

**Methods:**

Patients enrolled in studies GS-US-174-0102 and GS-US-174-0103 who had baseline liver biopsy–diagnosed cirrhosis and entered the open-label phase of the studies were included in the virologic and serologic analyses. Patients (both HBeAg positive and negative) with paired liver biopsies at baseline and 5 years (*N* = 348) were included in a histologic analysis.

**Results:**

After 5 years on study, comparing patients with and without baseline cirrhosis, respectively: 99.2 and 98.0 % achieved virologic response (hepatitis B viral load < 69 IU/ml) (*p* = 0.686); 79.7 and 81.9 % had normal serum levels of alanine aminotransferase (*p* = 0.586); 4.0 and 1.2 % developed hepatocellular carcinoma (*p* = 0.044). In HBeAg-positive patients with and without baseline cirrhosis, HBsAg loss occurred in 14.4 and 8.3 % of patients, respectively (*p* = 0.188). One HBeAg-negative patient had HBsAg loss.

**Conclusions:**

This represents the largest analyses to date of CHB patients with sequential liver biopsies demonstrating that treatment with TDF for up to 5 years is associated with favorable virologic, serologic, and histologic outcomes, regardless of baseline cirrhosis status. Notably, histologic improvement was observed in the majority of cirrhotic and noncirrhotic patients.

## Background

It is estimated that up to 40 % of patients with chronic hepatitis B (CHB) will progress to cirrhosis, liver failure, or hepatocellular carcinoma (HCC) [1]. In fact, hepatitis B infection may account for 30 % of cirrhosis cases and 50 % of HCC cases worldwide [2]. Because high serum hepatitis B virus (HBV) DNA levels are associated with increased risk of developing cirrhosis and HCC [3, 4], intervening with highly potent antiviral agents may alter the clinical course and prevent the development of these adverse liver-related outcomes [2, 5]. There are limited data regarding the impact of long-term antiviral therapy on clinical outcomes in cirrhotic CHB patients. However, over the past decade, studies of oral nucleot(s)ide analogs have suggested that reducing HBV viral load may prevent disease progression in CHB patients and, in a small patient series, reverse preexisting cirrhosis [6–9].

Published results from a combined cohort of two ongoing phase 3 studies of tenofovir disoproxil fumarate (TDF) in CHB patients (*N* = 641) have provided evidence that long-term HBV suppression can promote significant fibrosis regression and cirrhosis reversal in the majority of treated patients [10]. Of the 348 patients with paired liver biopsies at baseline and year 5, 87 % experienced histologic improvement (defined as a ≥ 2-point reduction in the Knodell necroinflammatory score without worsening of fibrosis) and 51 % experienced fibrosis regression [10]. Furthermore, 74 % of the 96 patients with baseline cirrhosis exhibited reversal of cirrhosis at year 5 [10]. Of these patients, all but one had Ishak fibrosis scores improved by ≥2 units, and 58 % had fibrosis scores improved by ≥3 units. Twenty-four (25 %) patients had no change in liver fibrosis. One (1 %) patient had worsening of fibrosis from baseline to year 5 (Ishak fibrosis score increase from 5 to 6). The question remains, however, whether long-term outcomes in CHB patients treated with TDF are influenced by patients’ baseline cirrhosis status. In this subanalysis of the two phase 3 studies, we report long-term clinical, virologic, serologic, and histologic outcomes in patients with and without baseline cirrhosis.

## Methods

### Study design

**Fig. 1:**
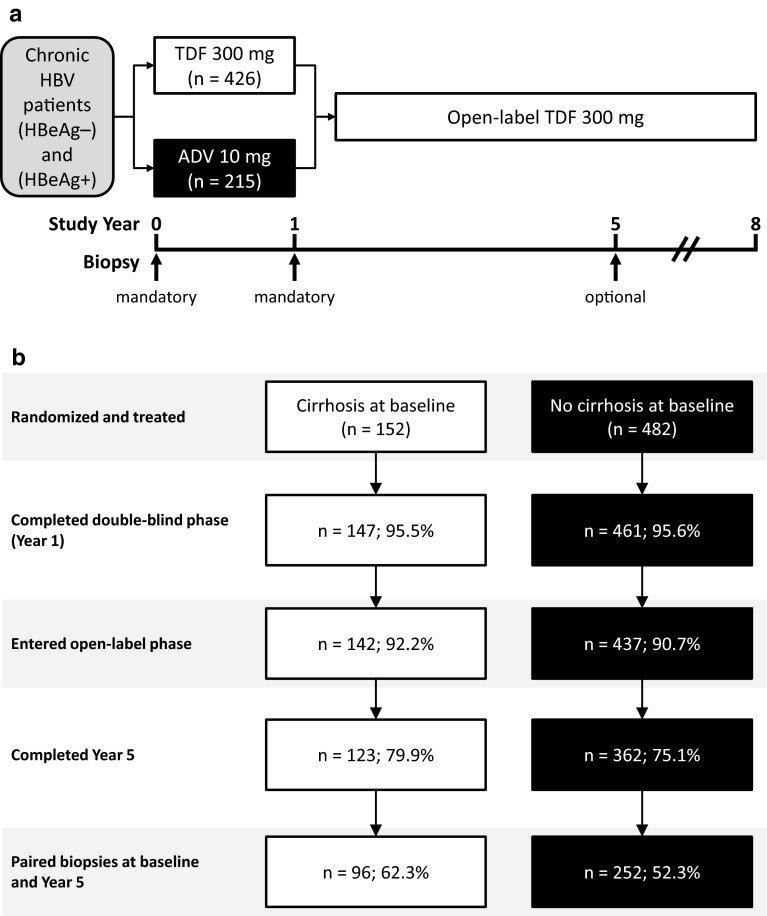
Study design and patient disposition. **a** The designs of the two phase-3 randomized double-blind placebo-controlled studies have been described previously [10–12]. Briefly, 641 patients with chronic HBV infection were randomized to tenofovir disoproxil fumarate (TDF) 300 mg daily (*n* = 426) or adefovir dipivoxil (ADV) 10 mg daily (*n* = 215) for 1 year. Afterward, patients were eligible to receive TDF 300 mg daily in an open-label extension study for up to 9 additional years. Mandatory liver biopsies were obtained at baseline and 1 year. A third, optional biopsy was obtained at year 5. **b** Of 641 patients randomized, 634 had liver biopsy data available at baseline. Of these 634, 152 were classified as having cirrhosis and 482 as having no cirrhosis. *HBeAg*− hepatitis B e antigen negative, *HBsAg*+ hepatitis B surface antigen positive, *HBV* hepatitis B virus

This retrospective pooled analysis investigates clinical, serologic, and histologic outcomes in a subset of CHB patients who had liver biopsy at baseline and year 5. These patients were initially treated for 1 year (48 weeks) with either adefovir dipivoxil (ADV) or TDF in two randomized, controlled phase 3 studies [GS-US-174-0102 (study 102) and GS-US-174-0103 (Study 103)] before continuing to open-label TDF for a planned additional 9 years. The studies are registered with ClinicalTrials.gov (NCT00117676 and NCT00116805, respectively). Six hundred forty-one patients were initially randomized; of these, 634 had liver biopsy data available at baseline. Complete medical history and full physical examination were done at baseline. Key exclusion criteria were coinfection with HIV-1, or hepatitis C or D virus, evidence of HCC, a creatinine clearance <70 ml/min, a hemoglobin level <8 g/dl, a neutrophil count <1,000/mm^3^, and liver decompensation or failure. Decompensated liver disease was defined as conjugated bilirubin >1.5 × the upper limit of normal (ULN), prothrombin time >1.5 × ULN, platelet count <75,000/mm^3^, serum albumin <3.0 g/dl, or prior history of hepatic decompensation (e.g., ascites, jaundice, encephalopathy, variceal hemorrhage). Detailed descriptions of the study populations, design, and methods have been reported previously [10–12] (Fig. 1a).

Demographic, baseline, and on-treatment characteristics of patients who had cirrhosis (Ishak fibrosis score ≥5) at baseline were compared with patients who did not have cirrhosis (Ishak fibrosis score ≤4) at baseline [13]. Measures of biochemical response included the proportion of patients with normal serum levels of alanine aminotransferase (ALT) and the proportion of patients with abnormal ALT levels at baseline who achieved ALT normalization (at or below the upper limit of normal (ULN), which was 34 IU/ml for females and 43 IU/ml for males). Serologic end points included serum hepatitis B surface antigen (HBsAg) loss and serum hepatitis B e antigen (HBeAg) loss. Virologic response was defined as the proportion of patients with plasma HBV DNA <69 IU/ml. The proportion of patients with plasma HBV DNA <29 IU/ml, the lower limit of quantification of the COBAS TaqMan assay, was also analyzed [10].

Patients who had paired liver biopsies at baseline and at year 5 were evaluated for histologic changes. The changes from baseline in Knodell necroinflammatory and Ishak fibrosis scores were compared between patients with and without cirrhosis. The procedure for evaluating liver biopsies has been previously described [10]. Regression of fibrosis was defined as a ≥1-point decline in the Ishak fibrosis score [10, 13].

### Statistical analyses

Descriptive statistics (mean and standard deviation for continuous variables, frequency and percentage for categorical variables) was used to summarize baseline demographics and disease characteristics for patients with and without baseline cirrhosis. Outcome measures at year 5 were summarized similarly using an on-treatment analysis, whereby all patients on study with non-missing data were analyzed. The Wilcoxon rank-sum test and Fisher’s exact test were used to compare continuous and categorical variables, respectively. Proportions of patients with HBsAg loss were estimated using the Kaplan-Meier method; comparison between groups was done via log rank test. Histologic improvements (Ishak fibrosis stage and Knodell necroinflammatory score) were compared using Fisher’s exact test on on-treatment data.

## Results

A total of 641 patients were randomized in the two phase-3 clinical trials (Fig. 1a). Of the original cohort, 91.3 % of patients (*n* = 585) entered the open-label phase, and 76.3 % (*n* = 489) completed 5 years of treatment.

### Baseline patient and disease characteristics

Baseline biopsies were obtained in 634 of 641 patients; of these, 152 (24.0 %) had cirrhosis at baseline and 482 (76.0 %) did not (Fig. 1b). Of the patients with baseline biopsies, 348 (54.9 %) also had biopsies at year 5 [10]. Biopsies were not performed in 141 patients at year 5, primarily because patients refused the procedure. Baseline characteristics of patients with and without cirrhosis are described below and summarized in Table 1. Of the patients with baseline cirrhosis, none were Child-Pugh B or C, none had a history of a decompensating event (e.g., hepatic encephalopathy, ascites), and none had two or more of the following laboratory abnormalities: INR ≥1.7, total bilirubin ≥2 mg/dl, or albumin ≤3.5 g/dl.

**Table 1:** Baseline demographic and disease characteristics

Factor	Cirrhosis at baseline (*n* = 152)	No cirrhosis at baseline (*n* = 482)	*p* value
Patients >40 years of age [*n* (%)]	104 (68.4)	218 (45.2)	<0.001
Male [*n* (%)]	123 (80.9)	345 (71.6)	0.026
Mean (SD) BMI (kg/m^2^)	*n* = 151 26.5 (4.6)	*n* = 479 25.1 (4.7)	<0.001
Asian [*n* (%)]	39 (25.7)	148 (30.7)	0.262
Genotype [*n* (%)]			0.043
A	34 (22.4)	67 (13.9)	
B	10 (6.6)	64 (13.3)	
C	27 (17.8)	83 (17.2)	
D	73 (48.0)	239 (49.6)	
Other^a^	8 (5.3)	29 (6.0)	
HBeAg positive [*n* (%)]	60 (39.5)	199 (41.3)	0.706
Previous lamivudine experience >12 weeks [*n* (%)]	20 (13.2)	55 (11.4)	0.566
Previous interferon experience [*n* (%)]	27 (17.8)	79 (16.4)	0.709
Mean (SD) HBV DNA (log_10_ IU/ml)	6.8 (1.43)	6.9 (1.51)	0.288
Mean (SD) HBsAg (log_10_ IU/ml)	3.9 (0.7)	4.1 (0.7)	0.017
Mean (SD) albumin (g/dl)	4.0 (0.4)	4.2 (0.3)	<0.001
Mean (SD) platelet count (cells/μl)	178,200 (50,350)	218,600 (54,460)	<0.001
Mean (SD) Knodell necroinflammatory score	9 (1.6)	8 (2.4)	<0.001
Ishak fibrosis stage [*n* (%)]			<0.001
0	0	1 (0.2)	
1	0	20 (4.1)	
2	0	248 (51.5)	
3	0	153 (31.7)	
4	0	60 (12.4)	
5	29 (19.1)	0	
6	123 (80.9)	0	
Patients with normal^b^ serum ALT levels [*n* (%)]	9 (5.9)	18 (3.7)	0.252

*ALT* alanine aminotransferase, *BMI* body mass index, *HBeAg* hepatitis B e antigen, *HBsAg* hepatitis B surface antigen, *HBV* hepatitis B virus, *SD* standard deviation

^a^The “other” category includes patients with genotypes E, F, G, and H and patients with no genotype information available

^b^The upper limit of the normal range (ULN) for ALT was 34 IU/ml for females and 43 IU/ml for males

#### Demographics

Statistically significant differences in age, sex, and body mass index (BMI) were observed at baseline between patients with and without cirrhosis. Patients with cirrhosis were more likely to be older than 40 years (68.4 vs. 45.2 %, *p* < 0.001) and male (80.9 vs. 71.6 %, *p* = 0.026) and to have a higher BMI (26.5 ± 4.6 vs. 25.1 ± 4.7 kg/m^2^, *p* < 0.001). Racial composition of both cohorts was similar; 25.7 % of patients with cirrhosis and 30.7 % of patients without cirrhosis were of Asian ancestry (*p* = 0.262).

#### Virologic and serologic characteristics

At baseline, genotype D was the predominant HBV genotype, detected in 48.0 % of patients with cirrhosis and 49.6 % of patients without cirrhosis. The cohort with cirrhosis had a higher proportion of patients with genotype A (22.4 vs. 13.9 %) and a lower proportion of patients with genotype B (6.6 vs. 13.3 %) than the cohort without cirrhosis (*p* = 0.043 for distribution of genotypes between cohorts). Mean levels of HBV DNA at baseline were similar between the groups. Patients with cirrhosis had lower mean quantitative HBsAg levels at baseline than patients without cirrhosis. There was no statistical difference in the percentage of patients with positive HBeAg status at baseline between cohorts with and without baseline cirrhosis (39.5 vs. 41.3 %, *p* = 0.706).

#### Clinical characteristics

Similar proportions of patients in both cohorts had normal serum ALT levels at baseline. Patients with baseline cirrhosis were more likely to have lower levels of serum albumin (*p* < 0.001) and lower platelet counts (*p* < 0.001) than patients who had no cirrhosis at baseline. Per protocol, all patients had compensated liver function at baseline.

### On-treatment factors

On-treatment factors for both patient groups are described below and summarized in Table 2.

**Table 2:** Clinical outcomes at 5 years, by baseline cirrhosis status (on-treatment analysis)

Factor	Cirrhosis at baseline (*n* = 152)	No cirrhosis at baseline (*n* = 482)	*p* value
HBV DNA <69 IU/ml [n/N (%)]	119/120 (99.2)	339/346 (98.0)	0.686
HBV DNA <29 IU/ml [*n/N* (%)]	119/120 (99.2)	337/346 (97.4)	0.465
Normal ALT level [*n/N* (%)]	94/118 (79.7)	286/349 (81.9)	0.586
Normalized ALT level [*n/N* (%)]	85/109 (78.0)	271/333 (81.4)	0.486
HBsAg loss^a,b^ (%)	14.4	8.3	0.188
Mean (SD) change in HBsAg from baseline to week 12 (log_10_ IU/ml)	*n* = 149 −0.1 (0.5)	*n* = 470 −0.2 (0.4)	0.037
HBeAg loss^a^ [*n/N* (%)]	26/42 (61.9)	54/119 (45.4)	0.075
Mean (SD) change in platelets (cells/μl)	*n* = 117 30,700 (43,930)	*n* = 343 20,100 (40,150)	0.030
Mean (SD) change in albumin (g/dl)	*n* = 119 0.3 (0.4)	*n* = 353 0.1 (0.3)	<0.001
Development of HCC [*n/N* (%)]	6/152 (4.0)	6/482 (1.2)	0.044

No patients in either group developed hepatic encephalopathy or variceal bleeding; one patient without cirrhosis at baseline developed ascites (in association with HCC)

*ALT* alanine aminotransferase, *HBeAg* hepatitis B e antigen, *HBsAg* hepatitis B surface antigen, *HBV* hepatitis B virus, *HCC* hepatocellular carcinoma, *SD* standard deviation

^a^HBeAg-positive patients only

^b^Kaplan-Meier estimated proportion

#### Virologic and serologic characteristics

After 5 years on study, nearly all patients in both cohorts achieved and maintained a virologic response, regardless of baseline cirrhosis status. At year 5, 99.2 % of the cohort with cirrhosis and 98.0 % of the cohort without cirrhosis had HBV DNA <69 IU/ml (*p* = 0.686); 99.2 % of the cohort with cirrhosis and 97.4 % of the cohort without cirrhosis had HBV DNA <29 IU/ml (*p* = 0.465). No patient in either cohort had persistent viremia, defined as never having had undetectable HBV DNA. Moreover, during year 5, only 4 of 495 patients experienced virologic breakthrough, 3 of whom had documented non-adherence to TDF.

Of the patients who were HBeAg positive at baseline, 61.9 and 45.4 % of patients with and without baseline cirrhosis, respectively, experienced HBeAg loss (*p* = 0.075) by year 5. Both cohorts demonstrated an early decline in circulating HBsAg; however, despite having a higher mean baseline HBsAg level, the mean change in HBsAg level at week 12 in the cohort without cirrhosis was greater than in the cohort with cirrhosis (*p* = 0.037). By year 5, HBsAg loss in the HBeAg-positive cohort occurred in 14.4 and 8.3 % of patients with and without cirrhosis, respectively (*p* = 0.188; on-treatment analysis; Kaplan-Meier estimate). Only one HBeAg-negative patient had HBsAg loss.

#### Clinical characteristics

The majority of patients achieved a biochemical response; 79.7 and 81.9 % of patients with and without baseline cirrhosis, respectively, had normal ALT levels (*p* = 0.586) at year 5. Greater increases in platelets (30,700 vs. 20,100 cells/μl; *p* = 0.030) and serum albumin (0.3 vs. 0.1 g/dl; *p* < 0.001) occurred in the cohort with cirrhosis, but both cohorts experienced significant improvements in these two parameters by year 5 on study. There were no episodes of liver decompensation, such as ascites or variceal bleeding, or liver failure observed in the two studies. Few patients in either cohort developed HCC at 5 years [cirrhotics: 4.0 % (*n* = 6) vs. noncirrhotics: 1.2 % (*n* = 6); *p* = 0.044]. At the time of this analysis, of the 12 patients who had been diagnosed with HCC, 3 died as a result of their disease, 4 with early-stage HCC achieved disease resolution via surgery or radiofrequency ablation, and 5 continued to have unresolved HCC [10].

#### Histologic characteristics

**Fig. 2:**
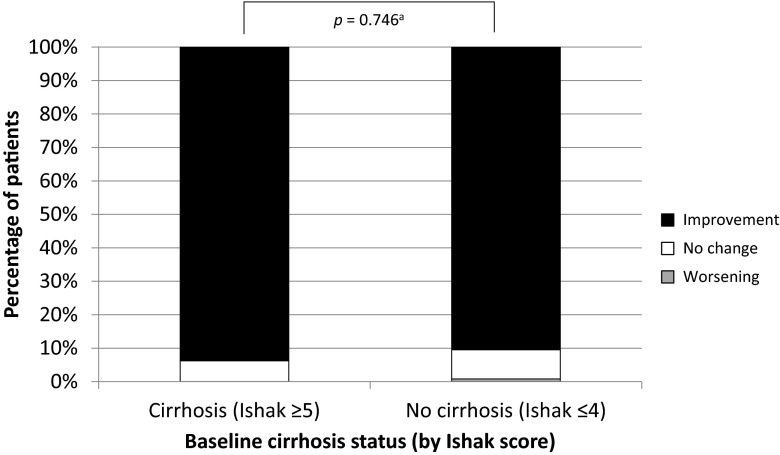
Histologic changes at year 5 among patients with and without baseline cirrhosis. Among 348 patients with paired biopsies at year 5 of treatment, 96 had baseline cirrhosis and 252 had no baseline cirrhosis. ^a^ *p* value calculated using Fisher’s exact test. Necroinflammatory response (Knodell scores) at year 5. Ninety-four percent of patients in the cohort with baseline cirrhosis and 91 % in the cohort with no baseline cirrhosis experienced improvement in the Knodell necroinflammatory score. Improvement was defined as a decrease in score of ≥1 unit; worsening was defined as an increase in score of ≥1 unit

The 5-year liver biopsy was obtained in 96 (63.2 %) of 152 patients with baseline cirrhosis and 252 (52.3 %) of 482 patients without cirrhosis. Among these, 93.8 % of patients with cirrhosis achieved an improvement in the Knodell necroinflammatory score of ≥1 unit compared with 90.5 % of patients without cirrhosis (*p* = 0.746) (Fig. 2). Only two (0.8 %) patients, both initially without cirrhosis, experienced a worsening of the Knodell necroinflammatory score. All other patients had no change in the necroinflammatory score.

### Safety

A summary of adverse events reported in patients with and without baseline cirrhosis through year 5 is provided in Table 3. There were no statistically significant differences between the two cohorts in key safety parameters. The incidences of renal adverse events in the cohort with and without baseline cirrhosis, as determined by an increase in serum creatinine >0.5 mg/dl above baseline (1.4 vs. 0.7 %), phosphorus levels <2.0 mg/dl (1.4 vs. 0.9 %), and creatinine clearance <50 ml/min (0.7 vs. 0.0 %), were all low. The single patient with baseline cirrhosis who experienced on-study creatinine clearance <50 ml/min had long-standing, medically managed hypertension, was >50 years of age, and was prescribed nonsteroidal antiinflammatory medications as needed.

**Table 3:** Safety summary during the open-label phase

Parameter [*n* (%)]	Cirrhosis at baseline (*n* = 142)	No cirrhosis at baseline (*n* = 437)
Patients with TEAE	122 (85.9)	358 (81.9)
Patients with TESAE	29 (20.4)	59 (13.5)
Patients with TEAE leading to study drug discontinuation	1 (0.7)	7 (1.6)
Patients with treatment-emergent grade 3/4 AE	21 (14.8)	56 (12.8)
Increase in serum creatinine of ≥0.5 mg/dl from baseline^a^	2 (1.4)	3 (0.7)
Serum phosphorus <2.0 mg/dl^a^	2 (1.4)	4 (0.9)
Creatinine clearance <50 ml/min^a,b^	1 (0.7)	0

The analysis set includes only patients who entered the open-label phase

*AE* adverse event, *TEAE* treatment-emergent adverse event, *TESAE* treatment-emergent serious adverse event

^a^Each parameter was confirmed on retest

^b^By the Cockcroft-Gault equation

## Discussion

Historically, CHB patients with cirrhosis have poorer prognosis, including greater risk for the development of HCC, than their CHB counterparts without cirrhosis [14, 15]. Our analyses suggest that, for the most part, long-term TDF treatment in CHB patients can lead to similar outcomes in patients with or without baseline cirrhosis. The exception is for HCC development, where our analyses revealed a significantly higher rate of HCC development in patients with baseline cirrhosis than without (4.0 vs. 1.2 %, *p* = 0.044). Nevertheless, the overall incidence of HCC development in either cohort was low. Rates of virologic response observed in our current analyses were similar between cohorts, suggesting that at year 5 in our studies, at least, the presence of cirrhosis at baseline may contribute more to the development of HCC during the study timeframe than viral load on therapy does. Therefore, surveillance for HCC should be performed regardless of HBV DNA suppression.

It has been demonstrated that decompensated cirrhosis is associated with poorer outcomes [16]. Moreover, HBV DNA suppression has been shown to prevent progression of clinical disease [8]. Because the two clinical studies excluded patients with decompensated liver disease, our analyses cannot address the effect of TDF on the clinical course of decompensated patients. However, over 5 years, there were no cases of hepatic decompensation in either study, an encouraging observation given the 2.7 % annual rate of liver decompensation previously observed in CHB patients [17].

In our analyses, patients without cirrhosis were more likely to experience early HBsAg declines of greater magnitude. This observation might be related to the significantly higher level of HBsAg that noncirrhotic patients had at baseline, a pattern that has been reported before [18]. A detailed report on the kinetics of HBsAg decline and loss in studies 102 and 103 was recently published, confirming a significant correlation between early decline and loss of HBsAg [19].

The generalizability of our findings to patients with cirrhosis may be limited to those with compensated disease given the patient population enrolled under the studies. In addition, the analyses reported were retrospective and therefore subject to the limitations inherent to retrospective analyses.

Through 5 years of treatment, no significant differences were observed between the two cohorts in the incidence of treatment-emergent adverse events. Importantly, the occurrence of renal adverse events was low in both cohorts. In summary, our analyses provide evidence that treatment with TDF up to 5 years is equally efficacious and safe in patients with CHB, regardless of whether patients with CHB are noncirrhotic or have well-compensated cirrhosis at baseline.

## References

[1] Lok AS (2002). Chronic hepatitis B. N Engl J Med.

[2] Brown A, Goodman Z (2012). Hepatitis B-associated fibrosis and fibrosis/cirrhosis regression with nucleoside and nucleotide analogs. Expert Rev Gastroenterol Hepatol.

[3] Chen CJ, Yang HI, Su J, Jen CL, You SL, Lu SN, Huang GT, Iloeje UH (2006). Risk of hepatocellular carcinoma across a biological gradient of serum hepatitis B virus DNA level. JAMA.

[4] Iloeje UH, Yang HI, Su J, Jen CL, You SL, Chen CJ (2006). Predicting cirrhosis risk based on the level of circulating hepatitis B viral load. Gastroenterology.

[5] Lok AS, McMahon BJ (2009). Chronic hepatitis B: update 2009. Hepatology.

[6] Dienstag JL, Goldin RD, Heathcote EJ, Hann HW, Woessner M, Stephenson SL, Gardner S, Gray DF, Schiff ER (2003). Histological outcome during long-term lamivudine therapy. Gastroenterology.

[7] Hadziyannis SJ, Tassopoulos NC, Heathcote EJ, Chang TT, Kitis G, Rizzetto M, Marcellin P, Lim SG, Goodman Z, Ma J, Brosgart CL, Borroto-Esoda K, Arterburn S, Chuck SL (2006). Long-term therapy with adefovir dipivoxil for HBeAg-negative chronic hepatitis b for up to 5 years. Gastroenterology.

[8] Liaw YF, Sung JJ, Chow WC, Farrell G, Lee CZ, Yuen H, Tanwandee T, Tao QM, Shue K, Keene ON, Dixon JS, Gray DF, Sabbat J (2004). Lamivudine for patients with chronic hepatitis B and advanced liver disease. N Engl J Med.

[9] Schiff ER, Lee SS, Chao YC, Kew Yoon S, Bessone F, Wu SS et al. Long-term treatment with entecavir induces reversal of advanced fibrosis or cirrhosis in patients with chronic hepatitis B. Clin Gastroenterol Hepatol 2011;9:274–276 10.1016/j.cgh.2010.11.040 21145419

[10] Marcellin P, Gane E, Buti M, Afdhal N, Sievert W, Jacobson IM, Washington MK, Germanidis G, Flaherty JF, Schall RA, Bornstein JD, Kitrinos KM, Subramanian GM, McHutchison JG, Heathcote EJ (2013). Regression of cirrhosis during treatment with tenofovir disoproxil fumarate for chronic hepatitis B: a 5-year open-label follow-up study. Lancet.

[11] Heathcote EJ, Marcellin P, Buti M, Gane E, De Man RA, Krastev Z, Germanidis G, Lee SS, Flisiak R, Kaita K, Manns M, Kotzev I, Tchernev K, Buggisch P, Weilert F, Kurdas OO, Shiffman ML, Trinh H, Gurel S, Snow-Lampart A, Borroto-Esoda K, Mondou E, Anderson J, Sorbel J, Rousseau F (2011). Three-year efficacy and safety of tenofovir disoproxil fumarate treatment for chronic hepatitis B. Gastroenterology.

[12] Marcellin P, Heathcote EJ, Buti M, Gane E, de Man RA, Krastev Z, Germanidis G, Lee SS, Flisiak R, Kaita K, Manns M, Kotzev I, Tchernev K, Buggisch P, Weilert F, Kurdas OO, Shiffman ML, Trinh H, Washington MK, Sorbel J, Anderson J, Snow-Lampart A, Mondou E, Quinn J, Rousseau F (2008). Tenofovir disoproxil fumarate versus adefovir dipivoxil for chronic hepatitis B. N Engl J Med.

[13] Ishak K, Baptista A, Bianchi L, Callea F, De Groote J, Gudat F, Denk H, Desmet V, Korb G, MacSween RN (1995). Histological grading and staging of chronic hepatitis. J Hepatol.

[14] El-Serag HB, Rudolph KL (2007). Hepatocellular carcinoma: epidemiology and molecular carcinogenesis. Gastroenterology.

[15] Goldstein ST, Zhou F, Hadler SC, Bell BP, Mast EE, Margolis HS (2005). A mathematical model to estimate global hepatitis B disease burden and vaccination impact. Int J Epidemiol.

[16] Hyun JJ, Seo YS, Yoon E, Kim TH, Kim DJ, Kang HS, Jung ES, Kim JH, An H, Yim HJ, Yeon JE, Lee HS, Byun KS, Um SH, Kim CD, Ryu HS (2012). Comparison of the efficacies of lamivudine versus entecavir in patients with hepatitis B virus-related decompensated cirrhosis. Liver Int.

[17] Chen YC, Chu CM, Yeh CT, Liaw YF (2007). Natural course following the onset of cirrhosis in patients with chronic hepatitis B: a long-term follow-up study. Hepatol Int.

[18] Seto WK, Liu K, Wong DK, Fung J, Huang FY, Hung IF, Lai CL, Yuen MF (2013). Patterns of hepatitis B surface antigen decline and hbv DNA suppression in Asian treatment-experienced chronic hepatitis B patients after three years of tenofovir treatment. J Hepatol.

[19] Marcellin P, Buti M, Krastev Z, de Man RA, Zeuzem S, Lou L et al. Kinetics of hepatitis B surface antigen loss in patients with HBeAg-positive chronic hepatitis B treated with tenofovir disoproxil fumarate. J Hepatol 2014;61(6):1228–1237. doi:10.1016/j.jhep.2014.07.019 10.1016/j.jhep.2014.07.019 PMC5976831 25046847

